# Treatment planning for MR-guided SBRT of pancreatic tumors on a 1.5 T MR-Linac: A global consensus protocol

**DOI:** 10.1016/j.ctro.2024.100797

**Published:** 2024-05-18

**Authors:** Guus Grimbergen, Hidde Eijkelenkamp, Louk M.W. Snoeren, Rana Bahij, Uffe Bernchou, Erik van der Bijl, Hanne D. Heerkens, Shawn Binda, Sylvia S.W. Ng, Christelle Bouchart, Zelda Paquier, Kerryn Brown, Richard Khor, Robert Chuter, Linnéa Freear, Alex Dunlop, Robert Adam Mitchell, Beth A. Erickson, William A. Hall, Paola Godoy Scripes, Neelam Tyagi, Jeremiah de Leon, Charles Tran, Seungjong Oh, Paul Renz, Andrea Shessel, Edward Taylor, Martijn P.W. Intven, Gert J. Meijer

**Affiliations:** a Department of Radiation Oncology, University Medical Center Utrecht, The Netherlands; b Department of Oncology, Odense University Hospital, Denmark; c Department of Clinical Research, University of Southern Denmark, Denmark; d Department of Radiation Oncology, Radboudumc, Nijmegen, The Netherlands; e Department of Radiation Oncology, Odette Cancer Centre, Sunnybrook Health Sciences Centre, University of Toronto, Toronto, Ontario, Canada; f Department of Radiation Oncology, HUB Institut Jules Bordet, Brussels, Belgium; g Radiation Oncology, ONJ Centre, Austin Health, Heidelberg, Victoria, Australia; h The Christie NHS Foundation Trust, Manchester, UK; i The Joint Department of Physics, The Institute of Cancer Research and The Royal Marsden NHS Foundation Trust, London, UK; j Department of Radiation Oncology, Medical College of Wisconsin, Milwaukee, WI, USA; k Department of Medical Physics, Memorial Sloan Kettering Cancer Center, New York, NY, USA; l GenesisCare, Darlinghurst, New South Wales, Australia; m Division of Radiation Oncology, Allegheny General Hospital, Pittsburgh, PA, USA; n Princess Margaret Cancer Center, University Health Network, Toronto, Ontario, Canada

**Keywords:** MR-guided SBRT, Pancreatic cancer, Treatment planning, Consensus protocol

## Abstract

**Background and purpose:**

Treatment planning for MR-guided stereotactic body radiotherapy (SBRT) for pancreatic tumors can be challenging, leading to a wide variation of protocols and practices. This study aimed to harmonize treatment planning by developing a consensus planning protocol for MR-guided pancreas SBRT on a 1.5 T MR-Linac.

**Materials and methods:**

A consortium was founded of thirteen centers that treat pancreatic tumors on a 1.5 T MR-Linac. A phased planning exercise was conducted in which centers iteratively created treatment plans for two cases of pancreatic cancer. Each phase was followed by a meeting where the instructions for the next phase were determined. After three phases, a consensus protocol was reached.

**Results:**

In the benchmarking phase (phase I), substantial variation between the SBRT protocols became apparent (for example, the gross tumor volume (GTV) *D*_99%_ ranged between 36.8 – 53.7 Gy for case 1, 22.6 – 35.5 Gy for case 2). The next phase involved planning according to the same basic dosimetric objectives, constraints, and planning margins (phase II), which led to a large degree of harmonization (GTV *D*_99%_ range: 47.9–53.6 Gy for case 1, 33.9–36.6 Gy for case 2). In phase III, the final consensus protocol was formulated in a treatment planning system template and again used for treatment planning. This not only resulted in further dosimetric harmonization (GTV *D*_99%_ range: 48.2–50.9 Gy for case 1, 33.5–36.0 Gy for case 2) but also in less variation of estimated treatment delivery times.

**Conclusion:**

A global consensus protocol has been developed for treatment planning for MR-guided pancreatic SBRT on a 1.5 T MR-Linac. Aside from harmonizing the large variation in the current clinical practice, this protocol can provide a starting point for centers that are planning to treat pancreatic tumors on MR-Linac systems.

## Highlights

• Worldwide collaboration between thirteen 1.5 T MR-Linac centers.
• Documentation of the wide variation in current practice of MR-guided pancreas SBRT.
• Development of a consensus protocol for MR-guided SBRT of pancreatic cancer.
• Treatment can already be harmonized with a simple set of dosimetric instructions.

## Introduction

MR-guided stereotactic body radiotherapy (SBRT) has been established as a novel and promising therapy for pancreatic tumors. Early clinical outcomes demonstrated that stereotactic treatment with prescribed doses of up to 50 Gy in five fractions (equivalent to a biologically effective dose for *α/β* = 10 (BED_10_) of 100 Gy) can safely be delivered while maintaining low acute toxicity rates [1], [2], [3], [4], [5], [6].

These high dose levels in a complex anatomical region make SBRT treatment planning for pancreatic tumors a complex task. There are currently notable differences between documented institutional protocols with regards to prescribed dose, organ at risk (OAR) constraints and safety margins, see Table 1 [2], [5], [7], [8], [9], [10], [11], [12], [13], [14], [15], [16]. However, even if these descriptions are identical in two institutions, there can still be nontrivial differences in treatment plans due to the fact that target coverage is sometimes compromised in favor of the strict dosimetric constraints of abutting radiosensitive digestive, biliary, and vascular structures [17], [18]. This means that additional clinical and technical variables influence the dose distribution outside the usual dosimetric considerations. Moreover, there is a wide range of expertise levels in centers that treat pancreatic tumors with MR-guided SBRT, mainly due to the dispersed and still ongoing adoption of MR-Linac systems around the world. This lack of a consensus planning protocol increases the risk of suboptimal treatment delivery and may hinder proper evaluation of the added value of MR-guided SBRT in these patient groups.

**Table 1:** Existing treatment planning protocols for MR-guided SBRT for pancreatic cancer, as reported in various studies. Prescribed dose is given as # fractions x fraction dose. OAR, organ at risk; GTV, gross tumor volume; PTV, planning target volume; dd, duodenum; st, stomach; sb, small bowel; c, colon.

	**Prescribed dose (Gy)**	**OAR constraints**^1^	**GTV-PTV** **margin (mm)**
Bohoudi et al. [7]	5x8 Gy	*D*1*cc <* 33 Gy; *D*20*cc <* 25 Gy	3 mm
Chuong et al. [12]	5x10 Gy	*D*0*.*03 *cc <* 40 Gy; *D*0*.*5 *cc <* 35 Gy (dd, st, sb)	3 mm
		*D*0*.*03 *cc <* 43 Gy; *D*0*.*5 *cc <* 38 Gy (c)	
Daamen et al. [15]	5x8 Gy	*D*0*.*5 *cc <* 35 Gy; *D*10*cc <* 25 Gy (dd, st, sb)	3 mm
		*D*_0_*_._*_5_*_cc_ <* 32 Gy (c)	
Hassanzadeh et al. [14]	5x10 Gy	*D*0*.*5 *cc <* 36 Gy	5 mm
Henke et al. [8]	5x10 Gy	*D*_0_*_._*_5_*_cc_ <* 35 Gy (dd, c)	5 mm
		*D*_0_*_._*_5_*_cc_ <* 33 Gy (st)	
		*D*_0_*_._*_5_*_cc_ <* 30 Gy (sb)	
Koay et al. [10]	5x10 Gy	*D*0*.*5 *cc* < 40 Gy; *D*1*cc <* 35 Gy; *D*2*cc <* 30 Gy	5 mm
Parikh et al. [5]	5x10 Gy	*D*_0_*_._*_5_*_cc_ <* 33 Gy	3 mm
Placidi et al. [9]	5x6-8 Gy	Not reported	3 mm
Rudra et al. [2]	5x10 Gy	Multiple (multi-center)	3 mm
Stanescu et al. [16]	5x6-8 Gy	Not reported	5 mm
Tyagi et al. [11]	5x10 Gy	*D*0*.*035 *cc <* 33 Gy; *D*5*cc <* 25 Gy (dd, st, sb)	5 mm
		*D*0*.*035 *cc <* 33 Gy; *D*5*cc <* 30 Gy (c)	
Yoon et al. [13]	Multiple	*D*0*.*5 *cc <* 35 Gy	5 mm

1 Confined to the reported constraints for duodenum (dd), stomach (st), small bowel (sb) and colon (c).

To address this issue, a consortium of centers with a 1.5 T MR-Linac was founded, with the aim to create a harmonized treatment planning protocol for five-fraction MR-guided SBRT for pancreatic cancer. The consortium comprised thirteen centers across North America, Europe, and Australia that are treating or planning to treat pancreatic cancer with MR-guided SBRT. There were considerable differences in relevant experience at the time the consortium was founded, with the top three centers each having treated over 100 patients. This work reports the outcome of this collaboration, which is a consensus protocol for treatment planning for pancreatic tumors on the 1.5 T MR-Linac.

## Materials & methods

### Planning and evaluation process

To reach a consensus, an iterative planning exercise was designed in which each center was asked to create a clinically acceptable treatment plan for two example cases, according to a set of instructions that became more prescriptive in each phase. Treatment planning was performed in Monaco v5.51.10/v5.51.11, the treatment planning software (TPS) of the 1.5 T MR-Linac (Elekta Unity, Elekta AB, Sweden). The centers performed treatment planning independently, and were not able to view each other’s results during planning.

The central element for reaching a consensus was the joint evaluation and discussion that followed each planning exercise, in which the instructions for the next phase were determined. These evaluation sessions were attended by physicists, clinicians, and radiotherapy technologists (RTTs) from each center. These discussions were centered around the results from previous studies, clinical experiences, and current protocols of individual centers. This iterative plan – evaluate cycle was repeated until a consensus protocol was reached.

After the first phase, the estimated delivery times of the treatment plans were also taken into consideration during the discussions. As surrogate for estimated delivery time, the number of monitor units (MUs) reported by the TPS was used. Due to the slightly different calibration setups used between centers, the reported MUs were normalized to the standard reference setup of 1 MU = 1 cGy measured at source-axis distance (SAD) of 143.5 cm, in 10 cm of water, with a field size of 10x10 cm^2^.

After consensus was reached, harmonization was also evaluated statistically by performing Bartlett’s test for homogeneity of variances between critical dose-volume histogram (DVH) parameters calculated in the treatment plans of the final phase and treatment plans of the initial phase. A statistically significant difference in variance was defined as *p <* 0*.*05.

### Case data

**Fig. 1:**
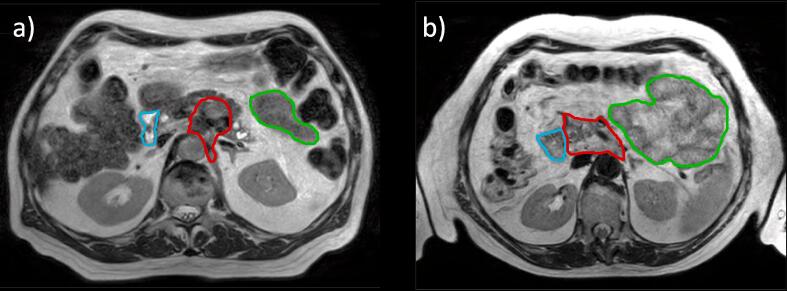
3D *T*_2_w MRI data of case 1 (a) and case 2 (b), on which the planning contours are projected (red = GTV, cyan = duodenum, green = small bowel). Note that not all contours are shown and that treatment planning itself was performed on CT imaging. (For interpretation of the references to colour in this figure legend, the reader is referred to the web version of this article.)

All participating centers were sent the same two anonymized data sets, reflecting two cases of locally advanced pancreatic cancer. Case 1 was a 62-year-old male with at least 10 mm distance between the tumor and the nearest luminal organs. This case was therefore considered to be favorable for radiotherapy planning (Fig. 1a). Case 2 was a 71-year-old female with the tumor abutting both the duodenum and small bowel along a considerable surface area, making this case less favorable (Fig. 1b).

Each data set consisted of a mid-ventilation CT (corresponding to the 20% phase of a 4DCT) and a structure set containing the gross tumor volume (GTV) and OARs. Treatment planning was performed on the CT scan to bypass differences in electron density conversion protocols. Centers were not allowed to edit existing OAR and target contours in the structure set, but were in phases I and II allowed to create custom planning target volume (PTV) and other margin structures (e.g. planning organ at risk volumes (PRVs)) and delineate additional OARs. To aid in this, auxiliary imaging was provided in the form of the complete 4DCT, arterial and portal phase contrast-enhanced CTs and a 3D *T*_2_-weighted MRI.

## Results

After three phases of planning and evaluation, a consensus protocol was reached within the consortium. The instructions for the three planning phases were as follows:

Phase I. Create a treatment plan according to the native five-fraction SBRT protocol of each center. This included the local dose prescription, OAR constraints, PTV/PRV margins, beam configuration, et cetera.
Phase II. Create a treatment plan adhering to the same consensus-based dose prescription and DVH constraints/objectives for the GTV, PTV, and most critical OARs, and PTV margin.
Phase III. Create a treatment plan using the same consensus-based template for the TPS. The TPS template not only contained the exact DVH constraints and objectives, but also dictated the specific cost function(s) for each planning structure, beam configuration, and sequencing parameters such as maximum amount of segments and minimum segment area. Treatment planning optimization was only allowed by modifying a fixed set of parameters of the preset cost functions. Modification of the structures, margins, and DVH constraints was not allowed.

**Fig. 2:**
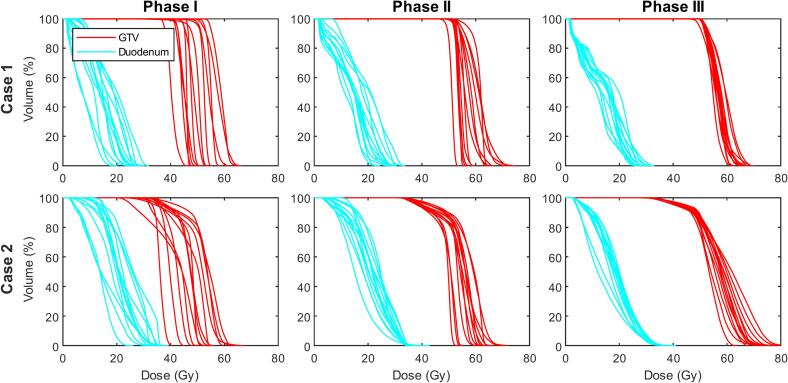
Dose-volume histograms (DVHs) of the GTV (red) and duodenum (cyan) from all thirteen centers. The DVHs are extracted from the treatment plans of case 1 (top row) and case 2 (bottom row), as created in phases I-III (columns). (For interpretation of the references to colour in this figure legend, the reader is referred to the web version of this article.)

**Fig. 3:**
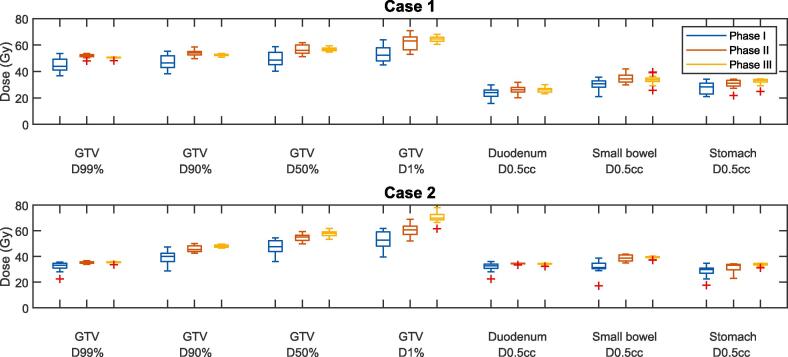
Boxplots of the distribution of the most critical DVH parameters of the GTV, duodenum, small bowel and stomach, in phases I-III. The whiskers of the boxplot extend to the minimum and maximum of the values not considered outliers (values further than 1.5 times the interquartile range from the box). The outliers are indicated by the red plus signs. (For interpretation of the references to colour in this figure legend, the reader is referred to the web version of this article.)

**Fig. 4:**
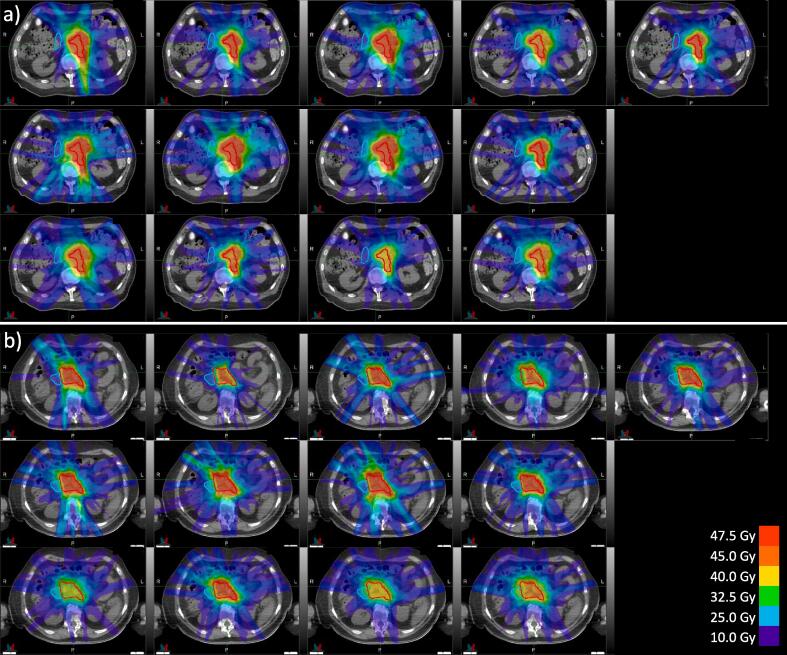
The dose distributions for a) case 1 and b) case 2 from all thirteen centers in phase I (variation in baseline practice). Red = GTV, cyan = duodenum. (For interpretation of the references to colour in this figure legend, the reader is referred to the web version of this article.)

The dosimetric results from the three planning phases are summarized in Fig. 2 and Fig. 3, where Fig. 2 contains the individual DVHs of the GTV and duodenum from all centers, and Fig. 3 contains boxplots of DVH parameters of the GTV (*D*_99%_, *D*_90%_, *D*_50%_, *D*_1%_), duodenum, small bowel, and stomach (all *D*_0_*_._*_5_*_cc_*). The individual dose distributions from all centers are shown in Fig. 4 for phase I, and supplementary Figs. S1 and S2 for phases II and III.

The sections below contain a detailed description of the instructions, outcomes, and discussion of each phase.

### Phase I

There was a considerable variation in the baseline practice among the consortium members, see Fig. 4 for all individual dose plans for case 1 and case 2. The main difference between the treatment protocols was the prescribed dose for the five-fraction protocol. The prescriptions were 5x7 Gy (n = 1), 5x8 Gy (n = 6), 5x9 Gy (n = 4), and 5x10 Gy (n = 5). For case 1, all centers were able to reach an acceptable level of target coverage as per the center-specific protocol, with the median (range) GTV *D*_99%_ = 44.0 Gy (36.8 – 53.7 Gy). Relative to the prescribed dose for each center, this is 103 % (94–110 %). This proved more challenging for case 2 due to the unfavorable anatomy, with the median (range) GTV *D*_99%_ = 33.1 Gy (22.6 – 35.5 Gy). Relative to the prescribed dose: 70 % (56–104 %). In this case, the loss in target coverage to adhere to the duodenum and small bowel constraint was also different between centers. As an illustration of the variance in OAR doses, the median (range) duodenum *D*_0_*_._*_5_*_cc_* was 24.0 Gy (15.7–29.8 Gy) for case 1, and 32.8 Gy (22.5–36.1 Gy) for case 2. Most centers created a dose gradient within the GTV boundaries. Differences in gradient steepness and its distance to the nearest OAR boundaries demonstrated different levels of conservative planning, and one center scaled down the prescribed tumor dose for case 2 from 5x8 Gy to 5x6 Gy (leading to a relative GTV *D*_99%_ of 104 %. If the original 5x8 Gy was the objective, the coverage would have been 88 %). Further differences were visible in dose homogeneity and conformity within the GTV (judged qualitatively), and high dose spillage outside the GTV into unspecified tissue.

Outside the dosimetric characteristics, the main noteworthy differences between protocols were the number of beams (range 8–19), PTV margins (range 3–5 mm), the exact definition of PTV objective criterion (in terms of minimum dose to minimum volume), dose grid resolution (range 2–3 mm^3^), Monte Carlo variance (range 1–3 %), and the use and size of planning organ at risk volume (PRV) and/or subtractive PTVs, meaning the PTV is locally shrunk to maintain a certain minimum distance to the nearest OAR. However, agreement was found on the use of abdominal compression, which was used by almost every center for passive mitigation of respiratory motion.

### Phase II

Considering the results from phase I, the consortium agreed on a basic set of goals and constraints to use for planning in phase II. It was decided to set a prescription dose of 5x10 Gy (GTV *V*_100%_ *>* 99 %), motivated by this dose level already being the standard in five centers and the favorable clinical outcomes reported in earlier studies [2], [5]. Consensus was also found in the OAR constraints for the critical luminal organs, with *D*_0_*_._*_5_*_cc_ <* 35 Gy for the duodenum, stomach, and colon, and *D*_1_*_cc_ <* 40 Gy for the small bowel. The more permissive constraint for the small bowel was motivated by the higher organ motility compared to e.g. the duodenum, and therefore a larger probability that the dose hot spot in the small bowel is in a different location from day to day. At tumor dose levels beyond 50 Gy, a constraint for the aorta, inferior vena cava, superior mesenteric artery, and celiac trunk of *D*_0_*_._*_1_*_cc_ <* 53 Gy was agreed upon. Furthermore, a GTV to PTV margin of 2 mm was chosen, anticipating the upcoming implementation of active motion management functionalities (gating and baseline drift correction) on the high-field MR-Linac [19]. Aside from real-time gating and baseline drift correction, this system also constantly monitors the real-time image registration quality and gates the beam if registration uncertainties arise. This ensures that the beam is only on when the GTV is inside the PTV within a high degree of confidence, allowing for tight PTV margins. PRVs were abandoned too for this reason, also motivated by the online adaptive treatment, and improved healthy tissue visibility compared to conventional (cone-beam CT-guided) radiotherapy. Finally, there was agreement to maximize the integral dose to the GTV within the above constraints.

The above instructions resulted in a substantial harmonization of treatment plans, with the median (range) GTV *D*_99%_ = 52.1 Gy (47.9–53.6 Gy) for case 1, and 35.4 Gy (33.9–36.6 Gy) for case 2 (Fig. 3). All OAR constraints were respected; the median (range) duodenum *D*_0_*_._*_5_*_cc_* was 26.2 Gy (20.1–31.7 Gy) for case 1, and 34.6 Gy (33.3–34.8 Gy) for case 2. The instruction to maximize the integral dose (without setting limits to the maximum dose) resulted in a much steeper dose gradient within the GTV, although deviations between plans became apparent in the median dose (*D*_50%_ range 51.1–61.9 Gy for case 1, 49.8–59.5 Gy for case 2) and maximum dose (*D*_1%_ range 52.8–70.8 Gy for case 1, 52.0–68.9 Gy for case 2). As a surrogate for treatment delivery time, the number of MUs for each plan was also evaluated for each treatment plan. Here, a considerable variation between centers was apparent, with the median (range) MUs were 2755 (1646–4057) for case 1, and 3578 (2146–4873) for case 2.

### Phase III

Following the harmonization of the most critical DVH parameters in phase II, the consortium adopted those planning constraints as part of the consensus protocol. One exception was the small bowel constraint of *D*_1_*_cc_ <* 40 Gy, which multiple centers deemed too permissive compared to existing protocols and literature. As such, the constraint was changed to *D*_0_*_._*_5_*_cc_ <* 40 Gy.

For phase III, a TPS template was created in which the agreed upon DVH parameters were set as planning constraints. The remaining technical settings within the template (cost functions, beam setup, sequencing parameters) were a selection of the most frequently used settings by the centers in the consortium, weighted by center experience. The template was distributed as a file set which centers were able to import into the TPS for phase III.

All centers were able to successfully create treatment plans using the consensus template. All but three centers deemed the resulting treatment plan clinically acceptable, with three centers citing that the abandoning of PRV would not be acceptable for current clinical use. Moreover, one of these centers cited an unacceptably high maximum dose within the GTV compared to their current practice. The DVHs (Fig. 2) and DVH parameters (Fig. 3) showed further harmonization of the treatment plans, with the median (range) GTV *D*_99%_ = 50.5 Gy (48.2–50.9 Gy) for case 1, and 35.6 Gy (33.5–36.0 Gy) for case 2. There was also a decreased spread in the median dose (*D*_50%_ range 54.8–59.5 Gy for case 1, 53.3–61.9 Gy for case 2). Although the maximum dose was overall increased, the spread did not become smaller compared to phase II (*D*_1%_ range 60.5–68.0 Gy for case 1, 61.5–78.0 Gy for case 2). For the duodenum *D*_0_*_._*_5_*_cc_*, the median (range) was 26.1 Gy (23.2–30.1 Gy) for case 1, and 34.5 Gy (32.3–34.6 Gy) for case 2. A statistical comparison between the DVH parameter variance of phase III showed a significantly reduced variation compared to phase I (*p <* 0*.*05) in all GTV parameters for case 1, and all GTV parameters except *D*_1%_ and OAR *D*_0_*_._*_5_*_cc_* for case 2. The detailed results of the statistical analysis are given in Table S1. The range in estimated MU count needed to deliver the treatments was also modestly decreased compared to phase II, with the median (range) MUs being 2398 (1979–2973) for case 1 and 3543 (3049–4551) for case 2. Finally, there was also harmonization observed in the isodose volumes (not confined to dose within a specific tissue or structure) of the treatment plans (see supplementary Fig. S3).

The final consensus protocol is summarized in Table 2. A detailed description of the technical parameters of the final consensus template is given in supplementary Table S2. The files to import the template directly in Monaco are available online through the following public download link: https://surfdrive.surf.nl/files/index.php/s/XxArrdkTk2sW4ru.

**Table 2:** Consensus protocol for five-fraction MR-guided SBRT for pancreas. Target objectives are subject to concession if OAR constraints cannot be met. There is no specific maximum dose limit to the GTV.

**Parameter**	**Value**
Prescription dose (#fractions x dose)	5x10 Gy
GTV	V100% *>*95 %
PTV	V95% *>*95 %
Duodenum, large bowel, stomach	*D*0*.*5 *cc <* 35 Gy
Small bowel	*D*0*.*5 *cc <* 40 Gy
Biliary duct	*D*0*.*1 *cc <* 50 Gy
Large vessels (aorta, inferior vena cava)	*D*0*.*1 *cc <* 53 Gy
Superior mesenteric artery	*D*0*.*1 *cc <* 53 Gy
Celiac trunk	*D*0*.*1 *cc <* 53 Gy
Kidneys	*D*_mean_ *<* 10 Gy; *D*67% *<* 16.8 Gy
Spinal cord	*D*0*.*1 *cc <* 28 Gy
GTV-PTV margin	2 mm with active motion management
Number of beams	9–14
Number of segments	As low as reasonably possible (lower limit = 45)
Passive motion management	Abdominal compression
Arms position	Arms down

## Discussion

Current clinical practices for MR-guided pancreas SBRT vary considerably between centers around the world. A consensus planning protocol has been developed for treating pancreatic tumors with MR-guided SBRT, reflecting a worldwide collaboration between thirteen 1.5 T MR-Linac users. Apart from the consensus protocol presented here, the discussions were considered especially fruitful across the whole consortium. The collective sharing, comparing, and benchmarking of treatment plans, protocols, and clinical experience was an exceptionally educational process for improving plan quality. With this work, we share these insights with the MRgRT community and provide a road map or guide for centers that are starting out with this complex treatment modality. As we found after phase II that treatments can largely be harmonized when using the same set of DVH constraints, this consensus protocol can easily be adopted by any MR-Linac center, both users of the high-field and low-field system. We have made the consensus template available for download. Centers may choose to adopt the complete template or parts thereof, or simply investigate outside clinical practice for educational purposes.

There were two main recurring points of debate, i.e. the attempt to maximize the dose to the GTV without considering any constraints here and the omission of PRVs. On these points, the final consensus was less outspoken than for the other aspects of the protocol, with three centers citing these omissions the reason they would not consider the resulting treatment plans clinically acceptable. It should be noted that two of these centers added the nuance that active motion management should at least be implemented before they would consider planning without PRVs. Indeed, recent efforts with the newly developed gating system for the 1.5 T MR-Linac have shown promising results in terms of congruence of the delivered and planned dose within the upper abdomen [19], which might change the future perspective on the general need for PRVs.

Furthermore, constraints like *D*_0_*_._*_1_*_cc_ <* 120 % for the PTV were part of conventional protocols to both force a homogeneous dose distribution and limit high dose to healthy tissue within the PTV. However, MR-guided radiotherapy has led to substantially smaller safety margins, decreasing the amount of healthy tissue within the high dose region. In combination with online motion management, and again anticipating active motion management, multiple centers within the consortium abandoned these constraints (the same argument can be made for the abandonment of PRVs). Moreover, the planning exercises in this study showed that an unbounded maximum dose can increase the mean tumor dose in cases where complete target coverage cannot be achieved. This might have a radiobiological advantage. On the other hand, increasing the maximum dose might lead to more MUs needed for the treatment plan. This was reflected in the relatively large range of MUs for case 2 after phase III (3049–4551), probably due to the large spread of GTV *D*_1%_ for this case (61.5–78.0 Gy), which was not reduced compared to phase II.

In an informal inquiry within the consortium shortly after this project ended, we found that almost all centers used or were planning to use a TPS template very similar to the consensus template from phase III. One center stated that they were not planning on adopting the template, but were looking into PTV and PRV margin reduction since joining this study.

In this study, target and OAR delineations were already provided. Although all centers noted that their delineation protocol is based on the SABR guidelines [20], it is known that delineations of pancreatic tumors and surrounding OARs is subject to nontrivial interobserver variation [21], [22]. This might contribute to further treatment plan differentiation in clinical practice. However, for the scope of this study, delineations were kept the same to make differences in planning strategy clearer between centers. This allowed us to come to a consensus in pure terms of treatment planning protocol only. Further harmonization of treatment should be focused on reducing interobserver variation, by creating consensus protocols for e.g. delineation guidelines and online imaging for treatment adaptation. The iterative plan evaluation framework from this study could very well be applied for these purposes as well.

The comparison between MUs in phases II and III lead to the valuable information that even if two plans are dosimetrically very similar, the estimated delivery time can still be very different. In the extreme case of our results (the case 1 MU range of 1979–2973), a treatment plan from one center could take more than twice as long to deliver in another center (assuming the same dose rate), which the latter center might find unacceptably long. Sequencing parameters which influence the number of MUs for a plan are often not exhaustively evaluated when centers design their treatment protocol, but we found that there is substantial room for optimization without loss of plan quality. Because these settings are embedded in the TPS template, phase III ensured harmonized plan sequencing and therefore the resulting MU range was smaller than in phase II. We should emphasize that with phase III, the primary goal regarding delivery time was once again dosimetric harmonization, and not necessarily achieving the *shortest* delivery time possible. This study only involved two example cases with different levels of dosimetric complexity, which is still the dominant factor in the amount of MUs. We therefore cannot guarantee that using the consensus template will lead to generalized optimal treatment times when adopted into clinical practice, but it will at least mitigate inter-user variability in radiation delivery time. The robustness of the template in an online setting can be evaluated in subsequent studies as the reference plan only serves as a starting point for daily plan adaptation. Such an evaluation will also help centers determining how they want to balance treatment time and plan complexity.

The OAR constraints in the final consensus protocol were based on collective experience, and the lack of trial-based evidence for clinical safety might be an objection against adoption of these constraints. However, there is a growing body of literature on clinical outcomes after MR-guided SBRT for pancreas. The recent SMART trial used the same 5x10 Gy dose prescription as our protocol, with very similar, albeit slightly more conservative constraints for the GI organs (duodenum, small bowel, stomach, and colon *D*_0_*_._*_5_*_cc_ <* 33 Gy) and reported no acute grade ≥ 3 toxicities that the authors deemed definitely related to treatment [5]. Moreover, the constraints in our protocol are below those identified in two dosimetric feasibility studies for dose escalated pancreas SBRT [10], [23]. We therefore consider these OAR constraints to be safe for adoption into clinical practice. The consensus protocol is subject to future refinement as the number of patients being treated on the 1.5 T MR-Linac increases globally and long-term outcomes data become available. Since most centers within this consortium are part of the prospective Multi-OutcoMe EvaluatioN of radiation Therapy Using the MR-linac (MOMENTUM) study (NCT04075305) [24], future clinical outcomes from a large cohort can be easily collected from centers that decide to adopt this protocol.

## Conclusion

A worldwide consortium of thirteen centers found a wide variation in treatment planning protocols for pancreatic tumors with MR-guided SBRT on a 1.5 T MR-Linac. After multiple rounds of internal discussions where protocols and treatment plans were compared, the consortium has developed a collective consensus protocol for MR-guided pancreas SBRT. Treatments can already be largely harmonized when the same basic set of DVH constraints and objectives is used. Further harmonization of both treatment planning and delivery is possible with the consensus TPS template, which is now available for download for all 1.5 T MR-Linac users.

## Funding

This work was supported by the Dutch Cancer Foundation (KWF) under Grant Agreement no. 12665.

## CRediT authorship contribution statement

**Guus Grimbergen:** Conceptualization, Methodology, Software, Writing – original draft. **Hidde Eijkelenkamp:** Formal analysis, Resources, Writing – review & editing. **Louk M.W. Snoeren:** Resources, Investigation, Writing – review & editing. **Rana Bahij:** Investigation. **Uffe Bernchou:** Investigation, Writing – review & editing. **Erik van der Bijl:** Investigation. **Hanne D. Heerkens:** Investigation, Writing – review & editing. **Shawn Binda:** Investigation. **Sylvia S.W. Ng:** Investigation, Writing – review & editing. **Christelle Bouchart:** Investigation, Writing – review & editing. **Zelda Paquier:** Investigation. **Kerryn Brown:** Investigation, Writing – review & editing. **Richard Khor:** Investigation, Writing – review & editing. **Robert Chuter:** Investigation, Writing – review & editing. **Linnéa Freear:** Investigation. **Alex Dunlop:** Investigation, Writing – review & editing. **Robert Adam Mitchell:** Investigation, Writing – review & editing. **Beth A. Erickson:** Investigation, Writing – review & editing. **William A. Hall:** Investigation, Writing – review & editing. **Paola Godoy Scripes:** Investigation, Writing – review & editing. **Neelam Tyagi:** Investigation, Writing – review & editing. **Jeremiah de Leon:** Investigation, Writing – review & editing. **Charles Tran:** Investigation. **Seungjong Oh:** Investigation. **Paul Renz:** Investigation, Writing – review & editing. **Andrea Shessel:** Investigation, Writing – review & editing. **Edward Taylor:** Investigation. **Martijn P.W. Intven:** Supervision, Resources, Writing – review & editing. **Gert J. Meijer:** Supervision, Resources, Funding acquisition, Writing – review & editing.
