# Supplementary material for: Treatment planning for MR-guided SBRT of pancreatic tumors on a 1.5 T MR-Linac: A global consensus protocol
Source: Clin Transl Radiat Oncol. 2024 May 18;47:100797. doi: 10.1016/j.ctro.2024.100797 (PMC11145226; doi:10.1016/j.ctro.2024.100797)
Supplement: Supplementary Data 1 [file mmc1.pdf]

Supplementary Materials

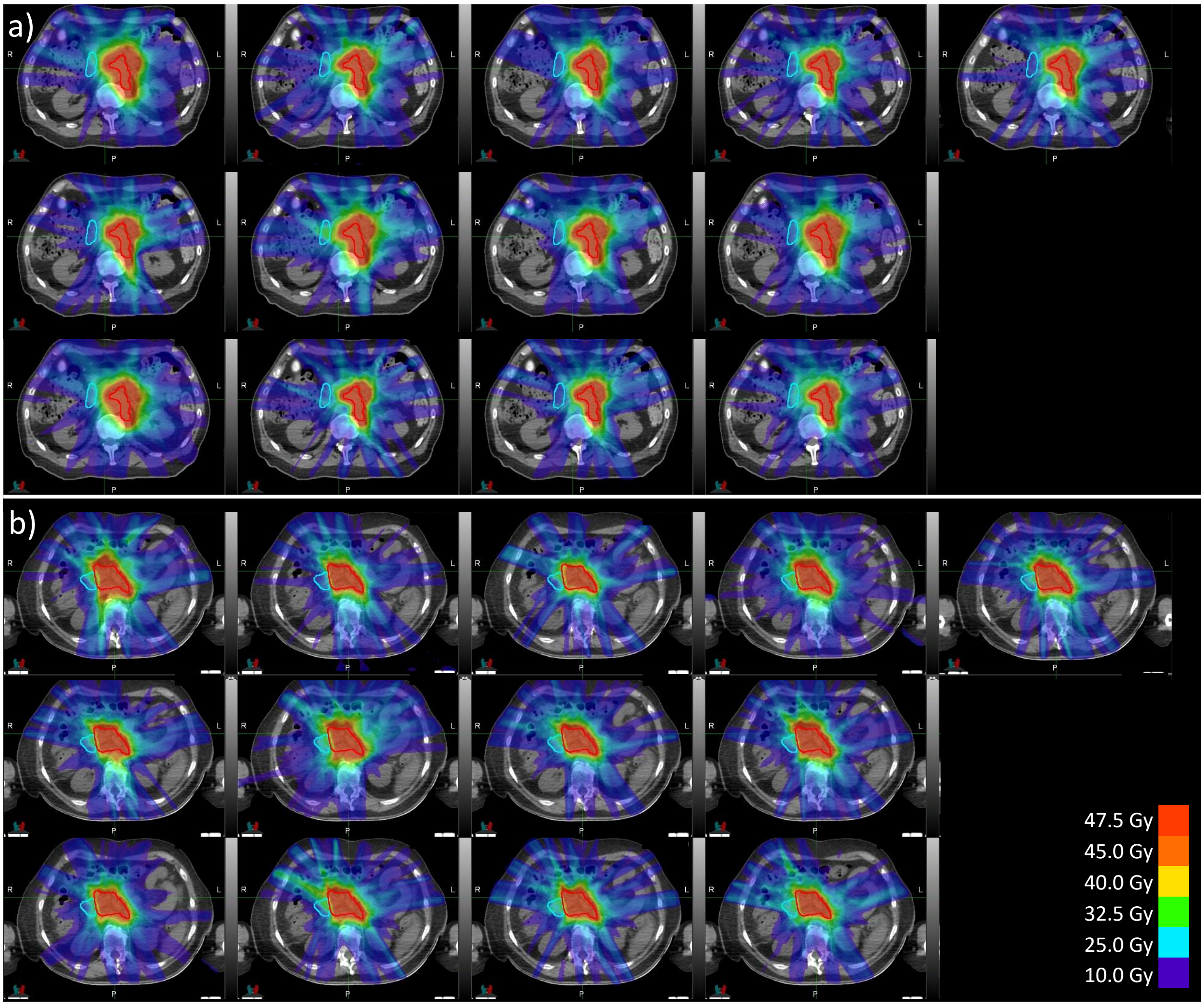

Figure S1: The dose distributions from all thirteen centers from phase II for a) case 1 and b) case 2. Red = GTV, cyan = duodenum.

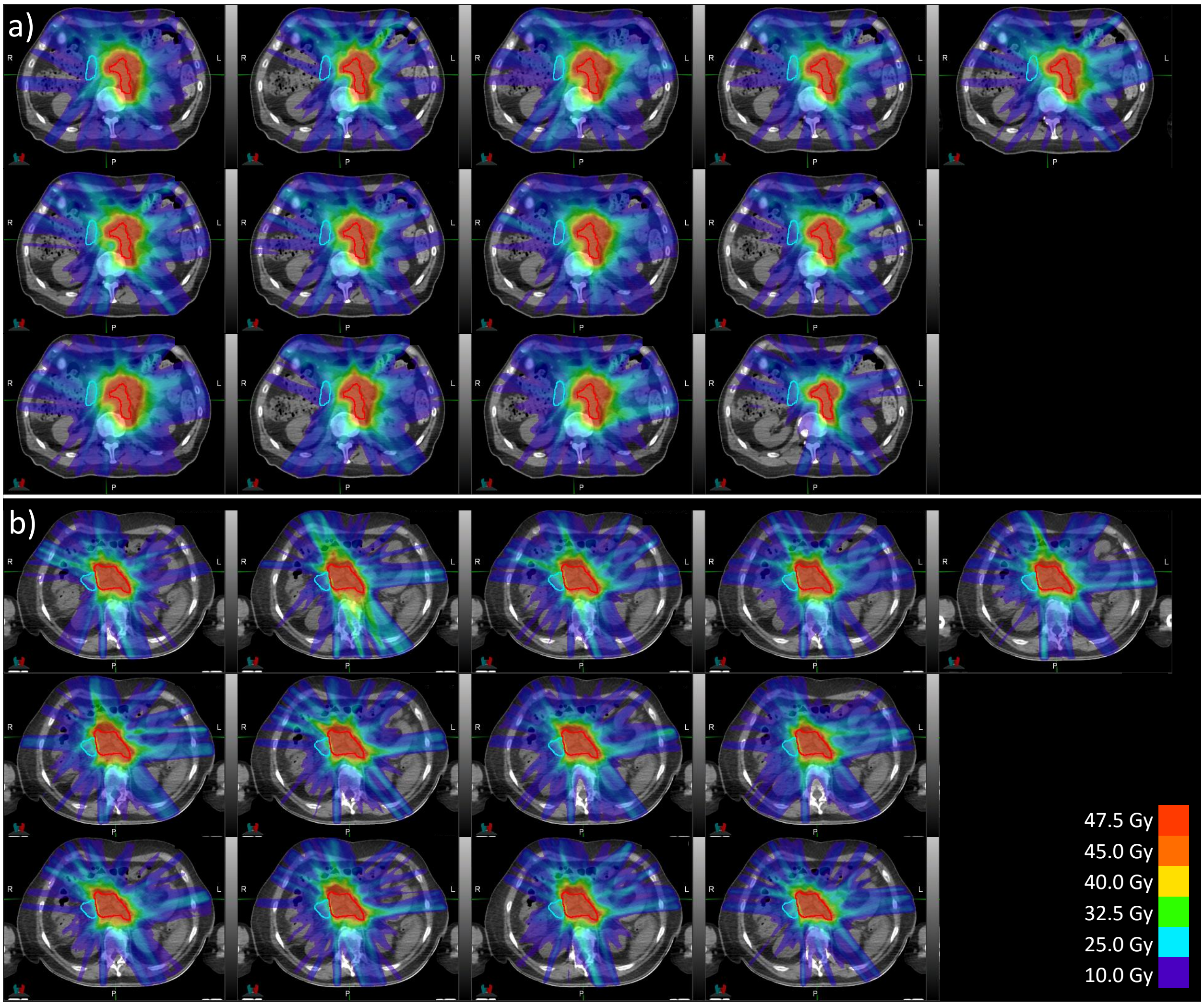

Figure S2: The dose distributions from all thirteen centers from phase III for a) case 1 and b) case 2. Red = GTV, cyan = duodenum.

Table S1: Results of Bartlett test of homogeneity of variances between the DVH parameters of phase III and phase I. p-values below 0.05 are considered statistically significant.

	**Case 1**		**Case 2**	
	K-squared	p-value	K-squared	p-value
GTV $D_{99\%}$	39.5	< 0.001	40.2	< 0.001
GTV $D_{90\%}$	33.2	< 0.001	23.9	< 0.001
GTV $D_{50\%}$	17.2	< 0.001	9.0	0.011
GTV $D_{1\%}$	10.2	< 0.01	2.9	0.232
Duodenum $D_{0.5cc}$	5.0	0.083	54.0	< 0.001
Small bowel $D_{0.5cc}$	0.3	0.845	26.5	< 0.001
Stomach $D_{0.5cc}$	3.5	0.174	19.7	< 0.001

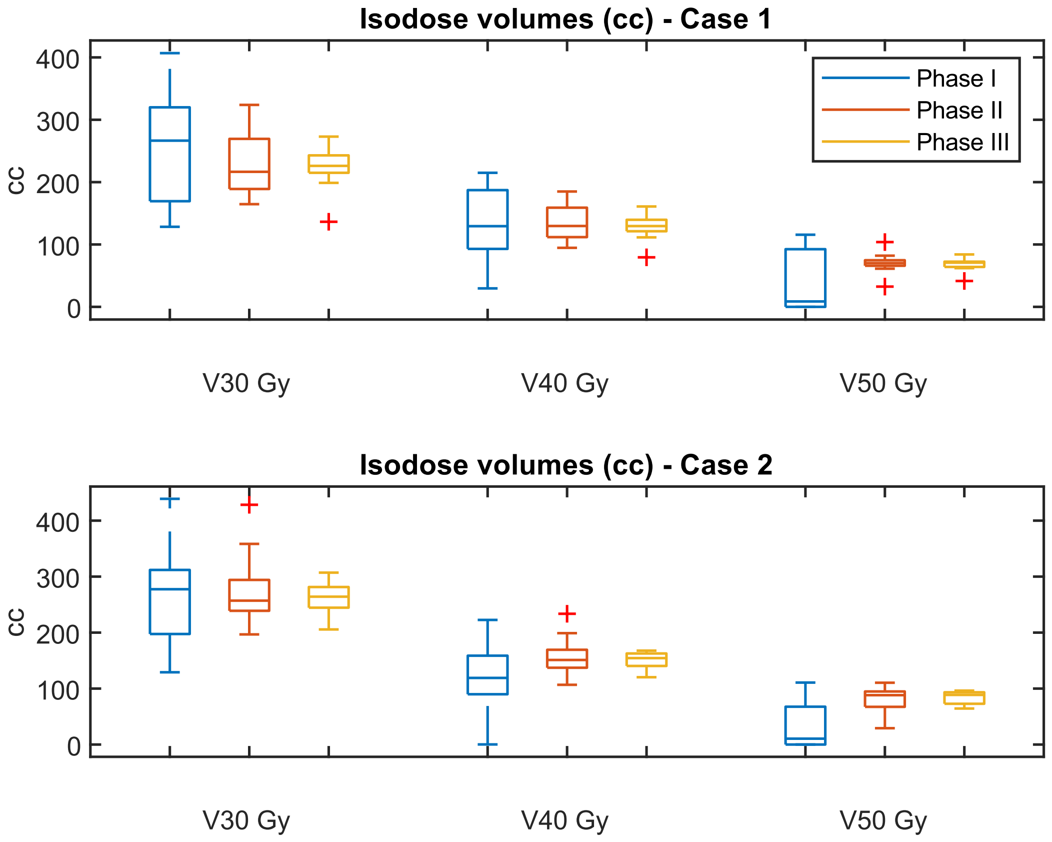

Figure S3: Boxplots of the volumes receiving at least 30 Gy, 40 Gy, or 50 Gy, in phases I-III. These were global isodose volumes, not confined to specific tissue. The whiskers of the boxplot extend to the minimum and maximum of the values not considered outliers (values further than 1.5 times the interquartile range from the box). The outliers are indicated by the red plus signs.

Table S2: Technical settings for the final treatment planning system (TPS) consensus template settings and parameters. The template is designed in Monaco (Elekta AB, Sweden) v5.51.10 (and has also been tested in v5.51.11), the TPS for the Unity MR-Linac. Please note that for some older versions of Monaco, care must be taken with the low number of segments as it might give errors during optimization due to a system bug. It is therefore strongly recommended to do rigorous offline testing when adopting the template.

**Parameter**	**Value**
Number of beams	14
Number of segments	45
Min. segment area	4.0 cm^2
Min. segment width	0.5 cm
Min. MU per segment	5 MU
Subtractive PTV-OAR margin	5 mm
GTV cost function	Target equivalent uniform dose (EUD)
PTV cost function	Target penalty
OARs cost function	Serial ($k = 20$)
Body contour cost function	Quadratic overdose
Evaluated OARs	Duodenum
	Small bowel
	Colon
	Stomach
	Spinal cord
	Aorta
	Celiac trunk
	Superior mesenteric artery
	Biliary duct
	Left kidney
	Right kidney
